# Systemic epigenetic response to recombinant lentiviral vectors independent of proviral integration

**DOI:** 10.1186/s13072-016-0077-1

**Published:** 2016-07-11

**Authors:** Tamas Aranyi, Daniel Stockholm, Roseline Yao, Catherine Poinsignon, Thibaut Wiart, Guillaume Corre, Nizar Touleimat, Jörg Tost, Anne Galy, Andràs Paldi

**Affiliations:** Université Evry Val d’Essonne, UMRS_951, Genethon, 91002 Evry, France; Ecole Pratique des Hautes Etudes, PSL Research University, UMRS_951, Genethon, 1 bis rue de l’Internationale, 91002 Evry, France; Inserm, U951, Genethon, 1 bis rue de l’Internationale, 91002 Evry, France; Genethon, 91002 Evry, France; Centre National de Génotypage, CEA-Institut de Génomique, 2, rue Gaston Crémieux, 91000 Evry, France

## Abstract

**Background:**

Lentiviral vectors (LV) are widely used for various gene transfer or gene therapy applications. The effects of LV on target cells are expected to be limited to gene delivery. Yet, human hematopoietic CD34+ cells respond to functional LVs as well as several types of non-integrating LVs by genome-wide DNA methylation changes.

**Results:**

A new algorithm for the analysis of 450K Illumina data showed that these changes were marked by de novo methylation. The same 4126 cytosines located in islands corresponding to 1059 genes were systematically methylated. This effect required cellular entry of the viral particle in the cells but not the genomic integration of the vector cassette. Some LV preparations induced only mild sporadic changes while others had strong effects suggesting that LV batch heterogeneity may be related to the extent of the epigenetic response.

**Conclusion:**

These findings identify a previously uncharacterized but consistent cellular response to viral components and provide a novel example of environmentally modified epigenome.

**Electronic supplementary material:**

The online version of this article (doi:10.1186/s13072-016-0077-1) contains supplementary material, which is available to authorized users.

## Background

The maintenance of a coordinated gene expression pattern is largely dependent on the chromatin organization in the cell nucleus. Epigenetic mechanisms such as DNA methylation play a key role in determining chromatin structure. In addition, these mechanisms represent an interface between environment and genome function [1].

Lentiviral vectors (LV) are convenient tools to achieve stable gene transfer in target cells for research, engineering or clinical applications such as gene therapy studies [2]. Integration-defective LV have also been designed for various transient gene delivery applications [3]. It is presumed that for all such uses, LV would not modify the function of transduced cells other than by effects of the transgene itself. Nonetheless several types of cellular responses have been caused by LV independently of the transgene. The proviral DNA was found to trigger transient innate immune responses in dendritic cells [4] and in mice [5] and its genomic insertion may potentially cause genotoxicity [2].

Recent studies have clearly demonstrated that LV can have a strong and long lasting epigenetic effects on transduced cells. For example, LV-s alone have been reported to epigenetically reprogram fibroblasts in culture independently of other external factors [6, 7]. We recently provided the first direct evidence that the in vitro transduction of hematopoietic stem and progenitor cells (HSC) with LVs induces DNA methylation of CpG nucleotides within 24 h after the first contact between the cells and vectors [8]. The epigenetic effects of LV have not been characterized in depth and it is not known if they are caused by the lentiviral provirus or by the viral components before integration of the viral genome.

The epigenetic alterations in HSC seem particularly important to assess given that self-renewal and differentiation of HSC are highly dependent on epigenetic mechanisms [9–13]. A potential impact of LV-induced epigenetic effects has to be considered when LV-s are used in gene therapy protocols based on LV-mediated gene transfer into patient-autologous CD34+ HSC to treat genetic diseases [2]. It is therefore important to investigate the exact nature of LV induced epigenetic changes in human HSCs in conditions relevant to gene therapy protocols.

The present study was conducted to determine the distribution of DNA methylation changes in the cellular genome in response to LV and the relation between them and the integration of the vector genome. We show that in addition to randomly occurring modifications a defined set of CpG islands associated to more than 1000 genes are systematically methylated. The DNA methylation changes occurred independently of the integration of the viral genome and only required cellular entry of the viral particle. Importantly, only some vector batches induce reproducible changes, other batches of the same vector produce only sporadic effects.

## Results

The Infinium Human Methylation 450K BeadChip (Illumina) provides a high resolution quantitative assessment of the CpG methylation level of relevant genomic regions. A major difficulty when comparing two methylation profiles with this method is to separate small but “true” changes from the background noise. Noise filtering can be performed intuitively by the use of pre-determine fixed threshold above which methylation changes are considered real. However, this traditional approach also eliminates small “true” changes. To overcome this difficulty, we have established an improved method for the detection of small methylation changes based on their repeated occurrence.

### New method for the analysis of the methylation changes using the Infinium 450K array

**Fig. 1:**
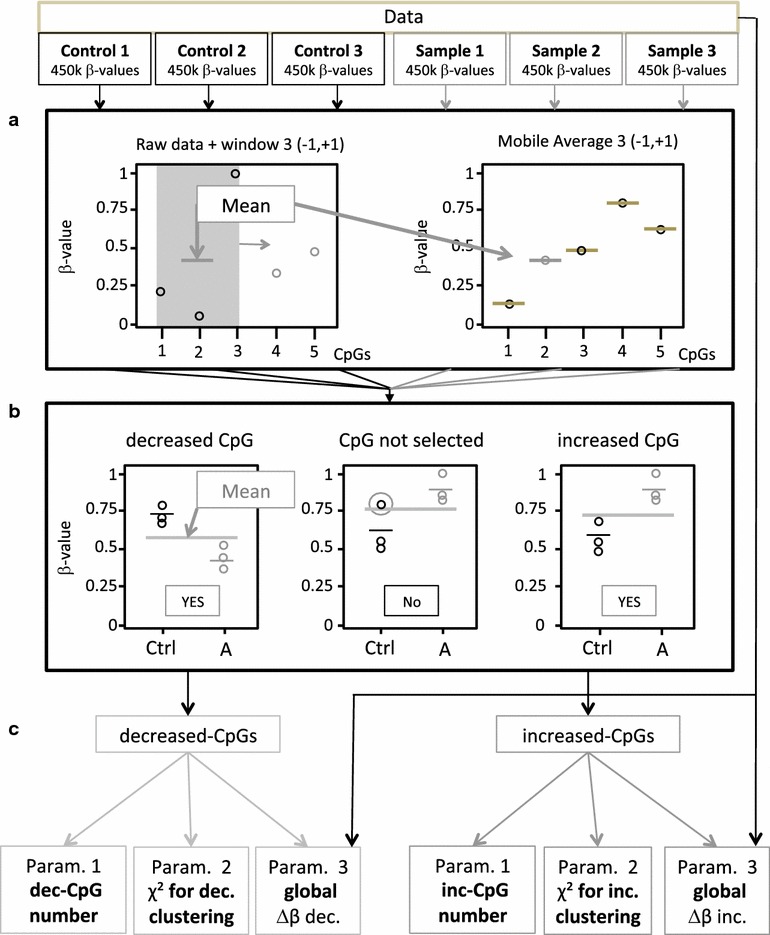
Workflow of data analysis and representation. The normalized *β*-values of triplicate samples and their corresponding controls are analyzed using the DAT algorithm as described in the text. **a** First, a moving average <*β*> is calculated for each CpG position in each sample and control. The average of all six sample <*β*> values is calculated ≪*β*≫. **b** The methylation level of a given CpG is considered as “increased” in the samples compared to controls if all the <*β*> values are higher then ≪*β*≫ or decreased if lower then ≪*β*≫. The above operation is repeated for all CpG-s analyzed by the Illumina chip. **c** Three parameters are calculated on the basis of the lists of “increased” and “decreased” CpG-s: (1) the total number of CpG-s (N); (2) chi2 calculated on the basis of the observed distribution of the CpG cluster sizes compared to the simulated random distribution of cluster sizes obtained with an identical number of CpG sites; (3) the median delta-*β* value of all the N CpG-s

A new algorithm called “double average technique” (DAT) was designed to identify very small, but reproducible differences between two series of samples after normalization of the raw data (see the workflow of the data analysis and representation on Fig. 1). This highly sensitive method identifies only reproducible differences in cytosine methylation level present in all samples compared to their reference controls. Sporadic differences are filtered. The method is implemented on triplicate experiments requiring the comparison of 3 samples with 3 corresponding controls. Thus, after hybridization on the 450K bead chip, 6 *β*-values are obtained for each CpG site tested. The sliding average (<*β*>) of the *β*-values of 3 consecutive CpG-s for each sample (*t*_1_, *t*_2_ and *t*_3_) and for each of the corresponding control (*c*_1_, *c*_2_ and *c*_3_) are calculated. The methylation of a given CpG site is considered to be different between the sample and control triplicates if all three values of the sliding averages of the samples are above (*t*_1_, *t*_2_, *t*_3_ ≥ increased methylation) or below (*t*_1_, *t*_2_, *t*_3_ ≤ decreased methylation) of the arithmetic average of the 6 <*β*>-values [(*t*_1_ + *t*_2_ + *t*_3_ + *c*_1_ + *c*_2_ + *c*_3_)/6] of the samples and controls. This constraint confers to the algorithm a very high stringency and allows the identification of all CpG sites with increased or decreased methylation independently of the extent of the difference and without applying the same fixed threshold to all CpG-s on the chip. The list of these CpG-s provides information on the total number of modified cytosines, their position in the genome and the extent of the increase or decrease of the methylation at each site.

Global assessment of the difference between the control and experimental triplicates is provided by three parameters. The first parameter (N) is the total number of CpG-s with altered methylation compared to controls. The second parameter shows the clustering of methylation changes in the genome. The value of this parameter is equal to the chi2 when comparing the distribution of the modified CpG-s to the theoretical random distribution of the same number of sites. The third parameter is the median difference between the *β*-values (delta-*β*) of the experimental and control triplicates for increased or decreased CpG-s. The three parameters were calculated separately for CpG-s with increased and decreased methylation.

### DNA methylation in CD34+ cells increases in response to LV transduction

First, we sought to determine if the previously observed changes in umbilical cord blood CD34+ cells using a Infinium 27K chip [8] could be confirmed with the Infinium 450K chip on a broader set of CpGs in CD34+ cells obtained from apheresed adult donors, a material more relevant to clinical use. In a pilot study we compared one of the samples examined in our previous report [8] with a DNA sample isolated from LV transduced CD34+ cells isolated from cytokine-mobilized human peripheral blood. We confirmed that CpG methylation changes in the cellular genome were induced in response to LV transduction in the CD34+ cells isolated from the two different sources (not shown). In all subsequent experiments we used CD34+ cells obtained from apheresed blood of adult individuals.

We then transduced CD34+ cells of 12 different donors using multiple batches of vector (Table 1) to examine the methylation profile of their genomic DNA using Infinium 450K chip runs. The characteristics of the vectors used and their potency is reported in Additional file 1. All 5 batches of LVs tested encoded GFP and were produced by transient transfection using second or third generation constructs. Three batches were concentrated by ultracentrifugation (LV1–LV3). In order to investigate the potential contribution of product contaminants, two batches (LV4–LV5) were purified and concentrated by ion exchange chromatography followed by ultrafiltration and gel filtration, a method that extensively removes protein and DNA contaminants from the vector preparation [14]. We analyzed the effects of several batches of non-integrating LV which were generated by completely removing the vector system HIV-1 integrase as a result of a stop codon in the *pol* gene (dINT) or by omitting the transfer plasmid leading to so-called “genome-empty” particles (dGEN). Two batches each of dINT and dGEN preparations were tested and verified to be unable to express a transgene and to integrate (Table 1). In addition, we also tested in these same experiments 3 batches of vectors unable to infect cells due to the omission of the envelope glycoprotein plasmid in the production process (dENV). All such defective vectors, except for the genome-deficient (dGEN) vectors included a transgene cassette coding for the green fluorescent protein (GFP). All were concentrated by ultracentrifugation (Additional file 1). They were tested in parallel with the fully functional LV using comparable amounts of particles on the basis of physical titer (Additional file 1).

**Table 1:** Effects of different batches of vectors and particles on CD34+ cells

	Integrating lentiviral vector LV					Integrase-deficient particles dINT		Transgene construct- deficient particles dGEN		Envelope-deficient particles dENV		
Batches tested	LV1	LV2	LV3	LV4	LV5	dINT1	dINT2	dGEN1	dGEN2	dENV1	dENV2	dENV3
Number of times tested independently	2	1	2	2	1	2	1	2	1	2	1	1
Number of different CD34+ cell donors tested	6	3	6	6	3	6	3	6	3	6	3	3
Average transduction efficiency (% GFP + cells ± SD) (range)	54 ± 8 (41–64)	52 ± 13 (39–7)	59 ± 6 (47–72)	57 ± 14 (39–85)	41 ± 7 (31–47)	0.1 ± 0.1 (0–0.3)	0.2 ± 0.2 (0–0.7)	0.1 ± 0.1 (0–0.1)	0 ± 0 (0–0.1)	0 ± 0 (0–0.3)	0 ± 0 (0–0.1)	0 ± 0 (0–0.1)
Average vector copy number per cell (VCN ± SD)	3.3 ± 1.1	NT	2.6 ± 1.3	0.4 ± 0.02	0.7 ± 0.1	0 ± 0	0.1 ± 0.1	0 ± 0	0 ± 0	0 ± 0	0 ± 0	0 ± 0
Increased methylation cluster effect^a^	Low, Low	High	High, High	Low, Low	Low	High, High	High	Low, High	High	Low, Low	Low	Low
Decreased methylation cluster effect^a^	Low, Low	Low	Low, Low	Low, Low	Low	Low, High	Low	Low, High	Low	Low, Low	Low	Low

^a^See Fig. 2b

Using a similar transduction protocol as described earlier [8], CD34+ cells were stimulated with early-acting cytokines for 20 h, cultured with LV (2 consecutive hits) during 24 h before genomic DNA was prepared for methylation analysis. Table 1 shows that all batches of functional LV resulted in similar transduction levels based on measures of the average vector copy number per cell (vcn) integrated and based on the percentage of cells expressing the GFP transgene after a short additional culture. No transduction was observed with the various defective vectors. Controls were set-up with cells from the same donors that were tested in parallel, cultured identically to the test cultures but without adding LV to the culture medium.

**Fig. 2:**
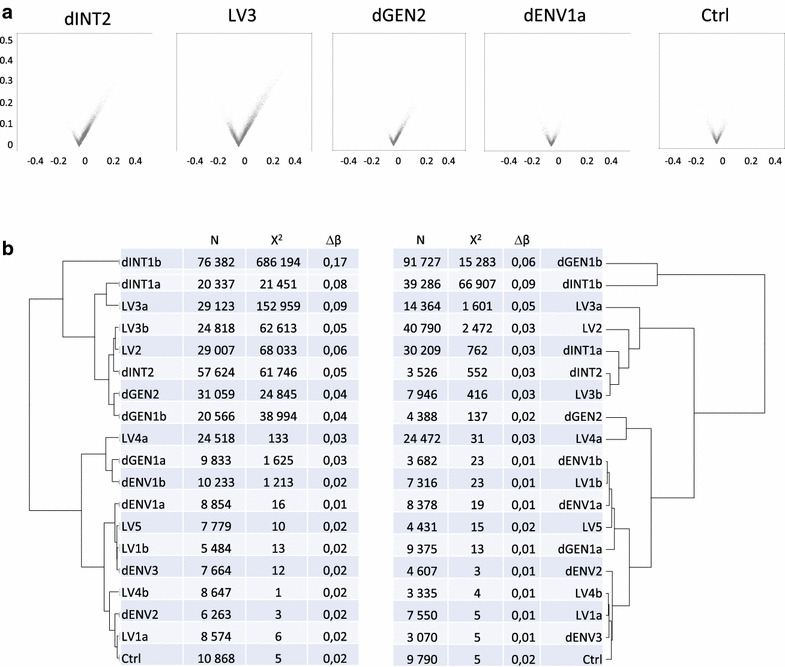
Analysis of DNA methylation changes. Analyses of 4 different chip runs (a, b, c, d) testing a total of 12 different batches of vector annotated accordingly (i.e. dENV1a and dENV1b indicates a repeat testing of dENV1 batch on chip a and chip b). **a** Volcano plots of representative experiments. From *left* to *right*: methylation differences observed in cells infected with an integrative lentiviral vector showing a high effect (LV2), integrase-deficient particles (dINT2), genome-deficient (dGEN2) and envelope-deficient (dENV1) particles as compared to control cells cultured but without any vector. The *last plot* on the *right* represents a control triplicate compared to another control triplicate (as in *bottom line* of **b**). *Each point* on a volcano plot represents the maximal delta-*β* value of a CpG site in a given triplicate as a function of the median of the three delta-*β* values of that triplicate. Only the CpG-s identified as displaying altered methylation between the samples and their corresponding controls are shown. *Points* representing CpG sites with increased and decreased methylation are displayed on the *right* and *left* sites of the plot. Note the higher number of points and higher median and maximal delta-*β* values with increased methylation in the first three samples. **b** Hierarchical classification of the experimental conditions depending on the extent of the increase (*left*) or the decrease (*right*) of the genomic DNA methylation in CD34+ cells. The classification was done using the three parameters indicated in the table: number of changed CpGs (N), chi2 of genomic distribution and median delta-*β*

The genomic DNA from the infected cells was extracted, bisulfite-converted, subjected to Infinium 450K array hybridization and analyzed using the DAT method. Volcano plot representation of the median and maximal delta-*β* values of CpG-s with increased and decreased methylation in triplicate samples of some representative experiments are shown on Fig. 2a. We calculated the three parameters (Fig. 1) that characterized the differences between the sample triplicates and their matched controls. In order to compare the effects observed with different vector preparations, we performed hierarchical clustering of the results. Data obtained for increased and decreased methylation were submitted to independent analysis (Fig. 2b).

When increased methylation is considered, the various LV-s segregated in two different clusters (Fig. 2b). The first cluster (“high”) included 2 of the 3 LV batches produced by ultracentrifugation and all of the integrase-deficient vectors and genome-deficient vector batches (Table 1). In addition to the relatively high number of methylated CpG-s, the distinguishing feature of methylation profiles in this group was the strong non-random distribution in the genome (high *χ*^2^) and relatively high proportion of the cells harboring methylation at these sites (high delta-*β*). The second cluster (“low”) included the 2 chromatography-purified and one ultracentrifuged batches of LV and all of the dENV batches. The “low” group also contained the background control of the experiments (Ctrl) that consists in a comparison between two sets of uninfected cell triplicates. The “low” group was characterized by higher dispersion of the modified CpG-s (lower *χ*^2^) and lower frequency in the cell population (lower delta-*β*). Repeated testing of the same LV batches provided the same categorization of their effects as seen for LV1, LV3, LV4.

Cluster analysis was also performed on the parameters for decreased CpG methylation. No clear segregation into two groups can be observed. The two batches (dGEN1 and dINT1) clustered separately from the others showing “high” effects on decreased methylation but this was not reproduced in a second independent experiment (Fig. 2b). Overall, the number of demethylated CpG-s and their incidence was often lower than the corresponding numbers for increased methylation in the same experimental conditions. This suggests that the cellular response to the different vectors involves principally de-novo methylation at a variable number of CpG sites and to variable extent. Nevertheless, some demethylation may also occur. The methylation changes are clearly unrelated to the integration of the viral genome in the host genome, because non-integrative vectors also induce methylation.

### The genomic localization of the changes shows recurrent methylation pattern

**Fig. 3:**
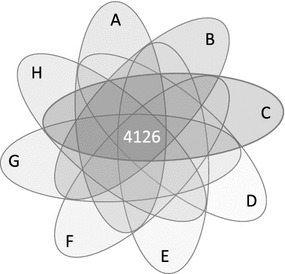
*Venn diagram* indicating the overlap between the eight different conditions identified as having “high” effect on increased methylation. 4126 is the number of CM-CpG sites. The different conditions were *A* dINT1b (76382 CpG-s); *B* dINTa (20337 CpG-s); *C* LV3a (29123 CpG-s); *D* LV3b (24818 CpG-s); *E* LV2 (29007 CpG-s); *F* dINT2 (57624 CpG-s); *G* dGEN2 (31059 CpG-s); *H* dGEN1b (20566 CpG-s), as defined on the Table 1

To further characterize the DNA methylation changes, we compared the lists of CpG-s with increased and decreased methylation in the “high” and “low” clusters. The lists in the “low” cluster did not share common CpG-s. Similarly, the lists of CpG-s with decreased methylation did not overlap significantly with one another. By contrast, the comparison of the 8 lists of CpG-s with increased methylation obtained in the “high” group revealed a substantial overlap. The methylation of 4126 CpG sites became higher in all 8 conditions that involved different vector types (Fig. 3), but also CD34+ cells from different donors (Table 1). The probability to get such a high number of identical changes fortuitously is essentially zero (*p* < 10^−60^). These sites represent 7–20 % of all CpG-s with increased methylation that were detected in the various conditions and almost 1 % of all sites present on the Illumina array. This high number of recurrently de novo methylated CpG-s (*commonly*-*modified CpG*-s, CM-CpG) in a subset of genes suggests that the exposure of the cells to vector particles triggers the action of a common mechanism.

**Fig. 4:**
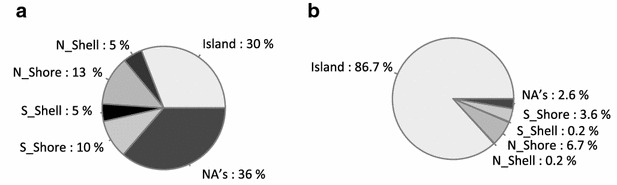
Genomic localization of the CM-CpG-s. **a** Distribution of all CpG-s interrogated by the array according to their sequence context as provided by the manufacturer. **b** Sequence environment of the CM-CpG-s

The majority (86 %) of the 4126 common modified CpG sites were found in CpG islands (CGI) (Fig. 4) in gene promoters, 5′UTRs, 1st exons and enhancers of 1059 genes (Additional file 2). The CpG-s in island “shores”, “shelves” or at non-assigned positions were strikingly underrepresented on our list (Fig. 4). The preferential localization of the CM-CpG-s to islands explains the high *χ*^2^ score obtained when the clustered distribution of the modifications were calculated (Fig. 2b).

We compared the list of the 1059 genes with that of genes reported to be expressed in CD34+ cells [15]. Of the 928 genes associated with CM-CpG-s and represented on the chip only 265 genes were expressed and 663 were silent in pre-activated CD34+ cells (the remaining 131 genes were not interrogated by the chip). This is significantly higher than the proportion of the non-expressed genes in the dataset (529 genes expressed versus 399 silent genes).

The integration of the HIV-1-derived LV-s in the host cell’s genome is not random. Although the DNA methylation changes occur independently of the integration, it is possible that the potential integration sites and the location of the CM-CpG-s are somehow related. To clarify this we calculated the distances between each CM-CpG and the position of closest integration site in a dataset of more than 30,000 LV integration sites [15]. The distribution of the distances was found to be random, showing that the sites targeted by the methylation changes and by the integration are unrelated.

In order to determine if these genes belong to specific functional categories, we have verified their functional ontology annotation using the DAVID tool. No significant enrichment was found. The 5 best represented functional categories of genes were involved in: regulation of transcription (GO:0045449; 224 genes, *p* < 7 × 10^−14^, 1.6 fold enrichment), regulation of RNA metabolic processes (GO:0051252; 185 genes, *p* < 1.1 × 10^−19^, 1.9 fold enrichment), regulation of transcription DNA dependent (GO:0006355; 184 genes, *p* < 2.4 × 10^−19^, 1.9 fold enrichment), positive regulation of biosynthetic processes (GO:0009891, *p* < 9.6 × 10^−11^, 2.1 fold enrichment) and neuron differentiation (GO:0030182, 77 genes, *p* < 4.7 × 10^−20^, 3.2 fold enrichment). Although any of these categories showed significant enrichment, examples of genes found on the list are known to impact the normal or pathological cell differentiation: genes coding for zinc finger-, homeobox-containing transcription factors, nuclear receptors,—de novo DNA methyl transferase Dnmt3A and SWI/SNF a chromatin structure modifying enzyme. Other genes on the list are implicated specifically in hematopoietic differentiation (e.g. GATA2, VENTX or CEBPd) or have important immunologic function (CD8a and b or BCL2L11). We also compared our gene list to the comprehensive cancer gene list established on the basis of observations from several laboratories (http://www.bushmanlab.org/links/genelists). Importantly, the two lists contained 152 genes in common. These observations clearly show that the methylated CM-CpG islands are not targeted for the biological function of the genes they regulate. Nevertheless, if the expression of these genes is perturbed by the methylation reprogramming, the functional consequences may be important and the differentiation of the cells may be impacted.

## Discussion

The purpose of the present study was twofold: to characterize the genomic distribution of the DNA methylation changes in response to LV and to investigate if these changes are related to the genomic integration of the vector. To this end, a stringent algorithm was developed to detect reproducible and clustered methylation changes. The main finding in this paper is that CD34+ cells respond to some batches of LV by systematic de novo methylation of the same CpG islands in the proximity of 1059 genes. The highly significant reproducibility of the “strong” effect in cells of 24 different donors and 8 different vector batches underscores the importance of the cellular response. However, other LV batches induce a milder “low” effect characterized by fewer modified CpG-s in dispersed genomic locations without recurrent changes and in a smaller proportion of cells. These observations can be understood as a heterogeneous manifestation of the same type of cellular response: some LV batches pass the threshold and induce “high” cellular reaction, while others stay below and elicit only a mild ‘low” effect. Although the precise mechanisms for the establishment of de novo methylation patterns in the genome are not yet elucidated [16–18], rapid changes are possible due to the highly dynamic maintenance of the DNA methylation [16, 18]. Various types of environmental stress are known to generate epigenetic response. For example, physiological stress related to under- or over-nutrition during fetal life is associated with global shifts towards DNA hypermethylation in CD34+ cells [19]. Stress related to in vitro culture of HSC-s also increases the methylation at specific sites in correlation with the loss of differentiation potential [13]. Our observations provide a new example of environmental stimulus or stress causing essentially de novo methylation.

An important issue is to evaluate the functional consequences of this response. In this respect, we observe that genes that become methylated belong to a wide range of functional pathways and not to specific categories. Therefore, these genes are probably not targeted for their function, but most likely because of their genomic location. Most of them are not expressed at the stage when the modification occurs, which reinforces this hypothesis. If the de novo methylation is maintained and perturbs gene expression at a later stage, the potential phenotypic effects are expected to be highly variable. This will make their study particularly difficult in future investigations.

The induced methylation changes were clearly independent of the integration of the vector genome in the cellular DNA and were not caused by proviral DNA because both the non-integrative dINT and dGEN vectors had the same effect. No difference in potency for CD34+ transduction or titer could distinguish the LV with “high” or “low” effects. Furthermore, the commonly methylated positions did not colocalize in a statistically significant manner with LV genomic integration sites reported in CD34+ cells by others [15]. We think that the observed changes represent an active cellular response to some vector components, but the identity of the trigger remains unknown. The HIV integrase is required for the steps of reverse transcription, for the formation of the preintegration complex and mediates genomic insertion by tethering the preintegration complex to the host cell chromatin and associated factors [20]. dINT particles which completely lack integrase due to a stop codon in the *pol* gene sequence exerted a strong DNA methylation effect on HSC which is therefore independent of any of all these steps described. The effect is also independent the accumulation of episomal LTR-circles which do not occur here. In some models, LV enhance chromatin remodelling and nuclear reprogramming via the stimulation of TLR3 pathway [6, 7]. It is unlikely that TLR3 is involved in our system, as double-stranded viral RNA that classically triggers TLR3 is not be produced significantly by dINT and dGEN vectors. Whereas we can exclude that the DNA methylation is caused by the integration of LV or by the provirus, there are other possibilities to consider based on the requirement for cell entry to generate the “high” cellular response. A single viral protein can cause genome-wide epigenetic changes as exemplified by adenovirus E1A [21, 22]. One possibility is that a minimal concentration of a viral component is necessary to induce the effect. A role of the VSVg envelope protein can be envisioned. VSVg is present on all LV tested even those with “low” effects but the number of VSVg molecules per particle are known to vary from 600 to 2200 per virion depending on the batches [23] and this could explain the variability of effects between LV batches. VSVg has been reported to constitute tubulovesicules in LV preparations that entrap plasmid DNA and induce TLR9-mediated innate immune responses in target cells [24]. In such case, standardized methods and the inclusion of a purification step should homogenize VSVg quality and quantity on LV and may be important to reduce a DNA methylation effect. Indeed, standardized LV preparations purified using ion exchange chromatography systematically induced “low” responses. Routine measurements of VSVg on LV batches will have to be developed to monitor this effect.

Another possible explanation is that the entry of large amounts of lentiviral vector components with non-integrative particles triggers the cellular response. All vector preparations, even infectious LV batches are heterogeneous, they contain varying levels of lentiviral protein components such as reverse transcriptase [23] and include no more than 1 % of infectious particles when comparing infectious and physical titers. Such inter-batch differences are not detected by infectivity measurements (Additional file 1). These non-integrative particles, when introduced in the cell, may trigger DNA methylation changes, as is observed with the dINT or dGEN. Additional studies will be needed to further address this point.

Altogether, our observations strongly suggest that LV batch quality is important, can be optimized at the upstream stages and may have a major impact on the transduced cells. Further studies are needed to develop quality control procedures to assess the DNA methylation effects of different preparations and to ensure that only LV with “low” properties would be used to transduce HSC.

LV-s are increasingly popular as gene transfer tools in the everyday laboratory practice, but also in therapeutic strategies where gene transfer is required. Integration-defective LV are also frequently preferred when transient expression of the transgene is required as, for example, in genome editing using CRISPR/Cas technology. Our observations which were made in clinically-relevant conditions (using apheresed CD34+ cells, the same cytokines and medium as used in clinical trials [25] and purified LV) point to the necessity of further investigating the mechanisms leading to the epigenetic changes. Understanding these mechanisms could open the way for further improving the safety of LV-s.

## Conclusions

Using a sensitive pipeline for data analysis we identified a previously uncharacterized but consistent cellular response to viral components that involves a systematic de novo methylation of more than 4000 CpG-s associated with 1059 different genes in the genome. These observations provide a novel example of environmental stress modified epigenome.

## Methods

### Cell culture

Human mobilized peripheral blood cells were obtained with informed consent according to international ethical principles and according to the French national bioethic law (no 2011-814). The study no DC-2012-1655 was approved by the French Ministry of Research and Higher Education-Bioethics office in September 2012. The anonymous samples were provided to us by accredited laboratories including a biological resource center (MYOBANK-AFM in Paris (http://www.biobanques.eu/ code BB-0033-00012), the French blood bank (EFS) (Hospital Henri Mondor, Créteil), the cell therapy laboratories of Gustave Roussy Institute (Villejuif, France) or University Hospital Center of Amiens (Amiens, France). The CD34+ haematopoietic stem cell fraction was purified by Ficoll gradient centrifugation and magnetic bead cell sorting according to the manufacturer’s instructions (Miltenyi). Purified cells were systematically analyzed by FACS and the CD34+ population was always >95 %. In total 12 purified samples from 12 different donors were used in the present study.

### LV transduction

CD34+ cells were transduced as described previously [8]. Briefly, after purification 1 × 10^6^ cells were pre-activated overnight at 37 °C in X-VIVO culture medium, supplemented with antibiotics and cytokines (Flt3-ligand (300 ng/ml), stem cell factor (300 ng/ml), trombopoietin (100 ng/ml) and IL3 (20 ng/ml). Transduction was performed in the presence of 4 μg/ml protamine sulfate (Sigma) with 5 × 10^7^ IG/ml (infectious genome) LV or equivalent amounts of p24 as specified in Additional file 1. A 2nd round of transduction was performed in the same conditions 6 h later. Transduction reaction was stopped 24 h after the beginning of the first round of transfection and cells were collected. After washing the cells a small fraction was put back to culture in preactivating culture supplemented with 10 % SVF and transfection efficiency was verified 8 days later by FACS of GFP+ cells. Integrated vector copy number was determined at the same time on the same fraction by qPCR.

### Lentiviral vectors

HIV-1 derived lentiviral vectors (LV) expressing eGFP under the control of the human PGK promoter and pseudotyped with VSVg using a 3- or 4-plasmid system as indicated in Additional file 1 were produced by transient transfection of 293T cells. The 3-plasmid system used the p8.74 plasmid coding for HIV-1 gag pol rev tat accessory genes. The 4-plasmid system used the pKLgagpol and pKRev plasmids. The culture medium was collected, concentrated by ultracentrifugation or chromatography and aliquots were stored at −80 °C. The chromatography-purified preparations were produced as described [14]. Vector titers were determined as infectious genome/ml (IG/ml) by qPCR after infection of HCT116 cells or as physical titers based on measured of P24 content by ELISA. Several deficient vectors were used in this study in addition to LV. dENV and dGEN vectors were respectively produced by transient transfection as above omitting the VSV-G encoding plasmid or the transfer plasmid encoding for eGFP. dINT particles were obtained by using the p8.74-INTSTOP plasmid in which a stop codon mutation was introduced in the pol gene. Titers of dINT, dGEN and dENV particles was determined by P24 content.

### Array based DNA methylation profiling and data analysis

LV transfection of the cells was followed by genomic DNA extraction. From each sample 500 ng genomic DNA was bisulfite treated using EZ DNA methylation kit and submitted to hybridization to the Infinium Human Mehtylation 450K BeadChip (Illumina) according to the manufacturer’s instruction. Results of the methylation assay were expressed as *β* values (ranging between 0—non-methylated, and 1—methylated) for each individual CpG present on the array. Raw data were extracted and normalized using Genome Studio. Only non-polymorphic CpG-s located on somatic chromosomes were considered. (The data are available under GEO accession number: GSE72867). We used the DAT algorithm to score individual sites for their methylation status. The DAT algorithm was implemented in “R”. The genes assigned to the CpGs were identified according to the array’s annotation. Functional annotation of the genes was performed using the online DAVID Functional annotation tool (http://www.david.abcc.ncifcrf.gov).

## Additional files

10.1186/s13072-016-0077-1 Characteristics of different batches of vector and particles tested on CD34+ cells.
10.1186/s13072-016-0077-1 Table with the names and ID-s of the genes associated with CM-CpG-s and the number of CM-CpG-s in the island.

## Abbreviations

LV: lentiviral vectors
HSC: hematopoietic stem and progenitor cells
DAT: double average technique
CM-CpG: commonly modified CpG-s
GFP: green fluorescent protein
HIV: human immunodeficiency virus
VSVg: vesicular stomatitis virus glycoprotein
vcn: vector copy number
