# Supplementary material for: Systemic epigenetic response to recombinant lentiviral vectors independent of proviral integration
Source: Epigenetics Chromatin. 2016 Jul 11;9:29. doi: 10.1186/s13072-016-0077-1 (PMC4940770; doi:10.1186/s13072-016-0077-1)
Supplement: Supplementary file 1 — 10.1186/s13072-016-0077-1 Characteristics of different batches of vector and particles tested on CD34+ cells. [file 13072_2016_77_MOESM1_ESM.pdf]

Supplemental Table 1: Characteristics of different batches of vector and particles tested on CD34+ cells

	**Integrating lentiviral vector LV**					**Integrase-deficient particles dINT**		**Transgene construct-deficient particles dGEN**		**Envelope-deficient particles dENV**		
Batches tested	LV1	LV2	LV3	LV4	LV5	dINT1	dINT2	dGEN1	dGEN2	dENV1	dENV2	dENV3
Production system (a)	Tri UC	Quadri UC	Tri UC	Quadri Chro	Quadri Chro	Tri UC	Tri UC	Tri UC	Tri UC	Tri UC	Quadri UC	Quadri UC
Transgene construct type (b)	pRRL	pCCL	pRRL	pCCL	pCCL	pRRL	pRRL	--	--	pRRL	pCCL	pCCL
Batch infectious titer (x E+08 IG/mL)	9	51	50	8. 1	6.2	--	--	--	--	--	--	--
Batch physical titer (x E+04 P24 ng/mL)	1.2	NT	7.36	0.48	0.62	0.3	5.8	4.6	68.4	2.2	31.1	33.7
Concentration tested (x E+08 IG/mL)	0.5	1	0.5	1	0.5	--	--	--	--	--	--	--
Concentration tested (P24 ng/mL)	667	--	735	591	384	667	667	667	667	667	667	667

(a) Tri = tri-transfection for tat-dependent second generation constructs using the p8.74 accessory plasmid or in the case of dINT batches, the p8.74INTstop mutated version; Quadri = quadritransfection for tat-independent third generation constructs using the pKLgagpol and pKRev accessory plasmids; UC= purified by ultracentrifugation; Chro = purified by ion exchange chromatography

(b) pRRL= pRRL PGK GFP WPRE; pCCL = pCCL PGK GFP WPRE

NT=not tested
