# Supplementary material for: Systemic epigenetic response to recombinant lentiviral vectors independent of proviral integration
Source: Epigenetics Chromatin. 2016 Jul 11;9:29. doi: 10.1186/s13072-016-0077-1 (PMC4940770; doi:10.1186/s13072-016-0077-1)
Supplement: Supplementary file 2 — 10.1186/s13072-016-0077-1 Table with the names and ID-s of the genes associated with CM-CpG-s and the number of CM-CpG-s in the island. [file 13072_2016_77_MOESM2_ESM.pdf]

Name	id	cg_num
SIM2	NM_005069	16
SOX2OT	NR_004053	14
HLA-J	NR_024240	14
NCRNA00171	NR_026751	14
HSPA1A	NM_005345	14
HSPA1L	NM_005527	14
TBX15	NM_152380	13
LMX1A	NM_177398	13
GNMT	NM_018960	13
SP5	NM_001003845	12
HLA-H	NR_001434	12
NR2E1	NM_003269	12
T	NM_003181	12
DPP6	NM_001039350	12
VENTX	NM_014468	12
ASCL2	NM_005170	12
TBX5	NM_000192	12
GFI1	NM_001127215	11
ACTA1	NM_001100	11
HPSE2	NM_001166244	11
PAX9	NM_006194	11
LBXCOR1	NM_001031807	11
ALX3	NM_006492	10
NR5A2	NM_205860	10
ZIC4	NM_032153	10
HIST1H3I	NM_003533	10
HCG4	NR_002139	10
PSMB8	NM_004159	10
WNT1	NM_005430	10
SPEG	NM_005876	9
FOXL2	NM_023067	9
C3orf72	NM_001040061	9
PROM1	NM_001145848	9
ZC3HAV1L	NM_080660	9
PRDM14	NM_024504	9
PAX2	NM_000278	9
PAX6	NM_001604	9
DGKZ	NM_201532	9
ME3	NM_006680	9
NKX2-1	NM_003317	9
FBXO39	NM_153230	9
HNF1B	NM_000458	9
TDRD10	NM_182499	8
SHE	NM_001010846	8
LOC100132215	NR_027069	8

TLX2	NM_016170	8
INHBB	NM_002193	8
HOXD9	NM_014213	8
TTLL3	NM_001025930	8
TRANK1	NM_014831	8
SFRP2	NM_003013	8
SCGB3A1	NM_052863	8
GDF6	NM_001001557	8
TLX1	NM_005521	8
LBX1	NM_006562	8
POU4F1	NM_006237	8
GSC	NM_173849	8
ABCC3	NM_001144070	8
FAM38B	NM_022068	8
UPK3A	NM_006953	8
PAX7	NM_013945	7
TMEM200B	NM_001003682	7
DMRTA2	NM_032110	7
TCTEX1D1	NM_152665	7
LHX4	NM_033343	7
C2orf70	NM_001105519	7
SIX3	NM_005413	7
HOXD3	NM_006898	7
PAX3	NM_181458	7
RASSF1	NM_170714	7
PARP14	NM_017554	7
FAM184B	NM_015688	7
UCP1	NM_021833	7
HLA-L	NR_027822	7
ETV7	NM_016135	7
PRR18	NM_175922	7
TMEM176B	NM_001101311	7
TMEM176A	NM_018487	7
PTPRN2	NM_002847	7
ESRP1	NM_001122826	7
GSDMD	NM_001166237	7
SPAG6	NM_172242	7
SPOCK2	NM_001134434	7
SLC18A2	NM_003054	7
SCUBE2	NM_020974	7
VWCE	NM_152718	7
ZNF785	NM_152458	7
SRCIN1	NM_025248	7
OVOL2	NM_021220	7
TCEA3	NM_003196	6
RUNX3	NM_001031680	6

COL9A2	NM_001852	6
NPR1	NM_000906	6
LHX9	NM_001014434	6
ESRRG	NM_001134285	6
ITPKB	NM_002221	6
LGALS8	NM_201544	6
TRIM71	NM_001039111	6
CWH43	NM_025087	6
GDNF	NM_199234	6
GPR150	NM_199243	6
TGFBI	NM_000358	6
TLX3	NM_021025	6
IRF4	NM_002460	6
NHLRC1	NM_198586	6
MEOX2	NM_005924	6
TRIL	NM_014817	6
TNFRSF10A	NM_003844	6
NKX2-6	NM_001136271	6
C8orf84	NM_153225	6
FOXE1	NM_004473	6
GPR120	NM_181745	6
FOXI2	NM_207426	6
WIT1	NR_023920	6
THY1	NM_006288	6
COL2A1	NM_001844	6
DHH	NM_021044	6
PTGDR	NM_000953	6
FES	NM_002005	6
MT1E	NM_175617	6
WNT3	NM_030753	6
TMC8	NM_152468	6
TMC6	NM_007267	6
COMP	NM_000095	6
LOC284798	NR_027092	6
GATA5	NM_080473	6
C22orf45	NR_028484	6
UPB1	NM_016327	6
LOC100128071	NM_001167676	5
TSSK3	NM_052841	5
CITED4	NM_133467	5
NPHS2	NM_014625	5
RASSF5	NM_182664	5
DNMT3A	NM_022552	5
MEIS1	NM_002398	5
LY75	NM_002349	5
PLA2R1	NM_001007267	5

TBR1	NM_006593	5
ADPRH	NM_001125	5
SEMA5B	NM_001031702	5
TRH	NM_007117	5
B3GALNT1	NM_001038628	5
PDGFRA	NM_006206	5
TLR2	NM_003264	5
SLC9A3	NM_004174	5
NPR3	NM_000908	5
CCL28	NM_148672	5
MYOZ3	NM_133371	5
HAND1	NM_004821	5
HLA-A	NM_002116	5
COL11A2	NM_080680	5
KCNK17	NM_031460	5
SLC22A16	NM_033125	5
WDR86	NM_198285	5
CRYGN	NM_144727	5
VIPR2	NM_003382	5
TNFRSF10C	NM_003841	5
APBB1IP	NM_019043	5
CXCL12	NM_001033886	5
SYT15	NM_031912	5
CYP26A1	NM_000783	5
RBP4	NM_006744	5
TLX1NB	NM_001085398	5
FLJ41350	NR_029380	5
HMX3	NM_001105574	5
CYB5R2	NM_016229	5
INSC	NM_001031853	5
RHOD	NM_014578	5
PHOX2A	NM_005169	5
RFX4	NM_213594	5
NKX2-8	NM_014360	5
FLJ44817	NM_001161498	5
DUOX1	NM_175940	5
ISLR2	NM_001130136	5
CYP11A1	NM_001099773	5
MPV17L	NM_001128423	5
MT1M	NM_176870	5
CA7	NM_001014435	5
ATP2A3	NM_174954	5
SLFN12L	NM_001145027	5
HCK	NM_002110	5
OLIG1	NM_138983	5
CLIC6	NM_053277	5

C2CD2	NM_015500	5
psiTPTE22	NR_001591	5
FAM19A5	NM_001082967	5
ESPN	NM_031475	4
PTPRU	NM_133178	4
TFAP2E	NM_178548	4
HPDL	NM_032756	4
PCSK9	NM_174936	4
LHX8	NM_001001933	4
C1orf51	NM_144697	4
KIAA1614	NM_020950	4
PKP1	NM_000299	4
LAD1	NM_005558	4
CSRP1	NM_004078	4
ATP2B4	NM_001001396	4
WNT3A	NM_033131	4
OBSCN	NM_052843	4
FAM150B	NM_001002919	4
OSR1	NM_145260	4
MFSD2B	NM_001080473	4
NOTO	NM_001134462	4
CD8A	NM_001145873	4
MAL	NM_022438	4
C2orf40	NM_032411	4
LOC440925	NR_027433	4
CCK	NM_000729	4
CACNA1D	NM_001128839	4
WNT5A	NM_003392	4
SLC12A8	NM_024628	4
ZIC1	NM_003412	4
RARRES1	NM_206963	4
VWA5B2	NM_138345	4
IDUA	NM_000203	4
EVC2	NM_001166136	4
DRD5	NM_000798	4
CD38	NM_001775	4
BANK1	NM_001127507	4
DDX60L	NM_001012967	4
IRX2	NM_033267	4
C5orf38	NM_178569	4
MARCH11	NM_001102562	4
PDE8B	NM_001029851	4
SQSTM1	NM_001142298	4
LOC729678	NR_027183	4
HLA-G	NM_002127	4
RNF39	NM_025236	4

TRIM15	NM_033229	4
LY6G5C	NM_025262	4
GLP1R	NM_002062	4
FAM162B	NM_001085480	4
TCF21	NM_198392	4
DACT2	NM_214462	4
DPY19L2P2	NR_027768	4
MNX1	NM_005515	4
EFCAB1	NM_001142857	4
SOX17	NM_022454	4
ADHFE1	NM_144650	4
FABP5	NM_001444	4
DPYS	NM_001385	4
FOXB2	NM_001013735	4
C1QL3	NM_001010908	4
C10orf114	NM_001010911	4
MKX	NM_173576	4
WDFY4	NM_020945	4
AGAP11	NM_133447	4
RNLS	NM_001031709	4
PPP1R3C	NM_005398	4
NKX1-2	NM_001146340	4
MACROD1	NM_014067	4
ATPGD1	NM_001166222	4
FGF19	NM_005117	4
C11orf88	NM_207430	4
ASAM	NM_024769	4
SYT10	NM_198992	4
B4GALNT1	NM_001478	4
LHX5	NM_022363	4
GJB6	NM_001110220	4
EFS	NM_005864	4
COCH	NM_001135058	4
SIX6	NM_007374	4
HEATR4	NM_203309	4
C14orf169	NM_024644	4
NGB	NM_021257	4
GATM	NM_001482	4
LOC145663	NR_033252	4
CRABP1	NM_004378	4
BNC1	NM_001717	4
LOC254559	NR_015411	4
MT1G	NM_005950	4
SLC16A11	NM_153357	4
TNK1	NM_003985	4
ANKRD13B	NM_152345	4

KRT19	NM_002276	4
STAT5A	NM_003152	4
LOC146880	NR_027487	4
MYO15B	NR_003587	4
HMHA1	NM_012292	4
GNA15	NM_002068	4
S1PR4	NM_003775	4
EVI5L	NM_145245	4
ACP5	NM_001611	4
EPHX3	NM_024794	4
RSPH6A	NM_030785	4
AVP	NM_000490	4
MAP1LC3A	NM_032514	4
KIAA1755	NM_001029864	4
TOX2	NM_001098796	4
TBX1	NM_080646	4
SEC14L4	NM_174977	4
AJAP1	NM_018836	3
RNF207	NM_207396	3
UBXN10	NM_152376	3
VWA5B1	NM_001039500	3
HPCAL4	NM_016257	3
FOXE3	NM_012186	3
ELAVL4	NM_001144777	3
ECHDC2	NM_018281	3
TMEM61	NM_182532	3
KANK4	NM_181712	3
GIPC2	NM_017655	3
BARHL2	NM_020063	3
C1orf194	NM_001122961	3
KIAA1324	NM_020775	3
PDE4DIP	NM_022359	3
ATP8B2	NM_020452	3
C1orf61	NM_006365	3
MIR9-1	NR_029691	3
DUSP23	NM_017823	3
KCNJ9	NM_004983	3
TDRD5	NM_173533	3
LAMC2	NM_018891	3
TRIM17	NM_016102	3
KIAA1383	NM_019090	3
SDCCAG8	NM_006642	3
CYS1	NM_001037160	3
NTSR2	NM_012344	3
CYP1B1	NM_000104	3
FIGLA	NM_001004311	3

DYSF	NM_001130981	3
CYP26B1	NM_019885	3
EMX1	NM_004097	3
RFX8	NM_001145664	3
DPP10	NM_001004360	3
PROC	NM_000312	3
HOXD10	NM_002148	3
FLJ32063	NR_026830	3
AOX1	NM_001159	3
NRP2	NM_201279	3
WNT6	NM_006522	3
MOGAT1	NM_058165	3
TWIST2	NM_057179	3
BOK	NM_032515	3
CAND2	NM_001162499	3
MLH1	NM_001167618	3
EPM2AIP1	NM_014805	3
P4HTM	NM_177938	3
ZMYND10	NM_015896	3
FEZF2	NM_018008	3
SHOX2	NM_001163678	3
ZNF141	NM_003441	3
SLC34A2	NM_006424	3
ZAR1	NM_175619	3
PITX2	NM_000325	3
FAT4	NM_024582	3
DCHS2	NM_001142552	3
C5orf49	NM_001089584	3
CRHBP	NM_001882	3
FLJ42709	NR_021490	3
TMED7-TICAM2	NM_001164468	3
TICAM2	NM_021649	3
SPOCK1	NM_004598	3
GFRA3	NM_001496	3
KCNIP1	NM_014592	3
SNCB	NM_003085	3
EIF4E1B	NM_001099408	3
NSD1	NM_172349	3
FLJ22536	NR_015410	3
HIST1H4L	NM_003546	3
HLA-F	NM_001098478	3
HLA-E	NM_005516	3
HLA-C	NM_002117	3
HLA-B	NM_005514	3
TCP11	NM_001093728	3
FOXP4	NM_001012427	3

RSPH9	NM_152732	3
KCNQ5	NM_001160133	3
SIM1	NM_005068	3
ULBP1	NM_025218	3
SLC22A3	NM_021977	3
RAMP3	NM_005856	3
WBSCR17	NM_022479	3
CLDN3	NM_001306	3
DLX5	NM_005221	3
MOGAT3	NM_178176	3
WNT2	NM_003391	3
IRF5	NM_001098631	3
DGKI	NM_004717	3
SHH	NM_000193	3
ADRB3	NM_000025	3
PENK	NM_006211	3
GRHL2	NM_024915	3
LHX2	NM_004789	3
EXD3	NM_017820	3
ALOX5	NM_000698	3
GPRIN2	NM_014696	3
DYDC2	NM_032372	3
DYDC1	NM_138812	3
CYP26C1	NM_183374	3
FRAT1	NM_005479	3
PSD	NM_002779	3
GFRA1	NM_001145453	3
EMX2OS	NR_002791	3
MGMT	NM_002412	3
TCERG1L	NM_174937	3
LRRC56	NM_198075	3
C11orf35	NM_173573	3
DBX1	NM_001029865	3
WT1	NM_024425	3
HRASLS5	NM_001146729	3
KCNK4	NM_033310	3
RASGRP2	NM_001098670	3
OVOL1	NM_004561	3
FGF4	NM_002007	3
MIR34B	NR_029839	3
BTG4	NM_017589	3
MIR34C	NR_029840	3
GLB1L3	NM_001080407	3
BCAT1	NM_005504	3
DDN	NM_015086	3
FAIM2	NM_012306	3

SLC26A10	NM_133489	3
ASCL1	NM_004316	3
TXNRD1	NM_001093771	3
HVCN1	NM_001040107	3
DNAH10	NM_207437	3
TMEM132C	NM_001136103	3
NBEA	NM_015678	3
TNFSF11	NM_003701	3
LMO7	NM_005358	3
MMP14	NM_004995	3
DHRS4L2	NM_198083	3
ADCY4	NM_139247	3
CLEC14A	NM_175060	3
VSX2	NM_182894	3
MLH3	NM_001040108	3
HHIPL1	NM_001127258	3
WDR72	NM_182758	3
IGDCC4	NM_020962	3
CHRNB4	NM_000750	3
LRRK1	NM_024652	3
SOX8	NM_014587	3
MMP25	NM_022468	3
PYCARD	NM_013258	3
MT3	NM_005954	3
B3GNT9	NM_033309	3
CPNE7	NM_153636	3
HIC1	NM_001098202	3
CLDN7	NM_001307	3
YBX2	NM_015982	3
NTN1	NM_004822	3
RASD1	NM_016084	3
ULK2	NM_014683	3
SLFN13	NM_144682	3
PPP1R1B	NM_032192	3
KCNH4	NM_012285	3
PTRF	NM_012232	3
WNK4	NM_032387	3
SOST	NM_025237	3
C17orf46	NM_152343	3
LOC100133991	NR_024434	3
MAPT	NM_001123067	3
LOC100130148	NR_024560	3
MIR152	NR_029687	3
COPZ2	NM_016429	3
B4GALNT2	NM_153446	3
DLX3	NM_005220	3

MYCBPAP	NM_032133	3
CA4	NM_000717	3
CYB561	NM_001915	3
LOC440461	NR_027283	3
CD300A	NM_007261	3
GALR2	NM_003857	3
SOCS3	NM_003955	3
FN3K	NM_022158	3
GNAL	NM_001142339	3
CIDEA	NM_198289	3
DSG2	NM_001943	3
KLHL14	NM_020805	3
MADCAM1	NM_130760	3
PTPRS	NM_130853	3
NFIX	NM_002501	3
LYL1	NM_005583	3
ANKLE1	NM_152363	3
KIRREL2	NM_199180	3
ARHGEF1	NM_004706	3
ZNF702P	NR_003578	3
ZNF665	NM_024733	3
ZNF542	NR_024056	3
ZNF154	NM_001085384	3
CPXM1	NM_019609	3
NKX2-2	NM_002509	3
VSX1	NM_014588	3
COL9A3	NM_001853	3
NKAIN4	NM_152864	3
FLJ16779	NR_024389	3
MIR155HG	NR_001458	3
OLIG2	NM_005806	3
CLDN5	NM_001130861	3
AIFM3	NM_001018060	3
SMTN	NM_006932	3
PDGFB	NM_002608	3
FAM83F	NM_138435	3
FAM109B	NM_001002034	3
LOC100271722	NR_027036	3
TYMP	NM_001113756	3
TP73	NM_005427	2
WDR8	NM_017818	2
PLEKHG5	NM_020631	2
MST1P9	NR_002729	2
ARTN	NM_057160	2
RNF220	NM_018150	2
TAL1	NM_003189	2

GPX7	NM_015696	2
PODN	NM_153703	2
C1orf87	NM_152377	2
C1orf173	NM_001002912	2
LRRC8D	NM_018103	2
C1orf59	NM_001102592	2
GSTM3	NR_024537	2
ADORA3	NM_001081976	2
S100A6	NM_014624	2
PEAR1	NM_001080471	2
KLHDC9	NM_001007256	2
RCSD1	NM_052862	2
FAM163A	NM_173509	2
PTPN7	NM_080588	2
PPFIA4	NM_015053	2
ACP1	NR_024080	2
SH3YL1	NM_015677	2
HPCAL1	NM_002149	2
HAAO	NM_012205	2
ATP6V1B1	NM_001692	2
LBX2	NM_001009812	2
SLC5A7	NM_021815	2
MALL	NM_005434	2
SCTR	NM_002980	2
GAD1	NM_000817	2
EVX2	NM_001080458	2
HOXD4	NM_014621	2
FRZB	NM_001463	2
CCDC140	NM_153038	2
SLC16A14	NM_152527	2
NPPC	NM_024409	2
PLCD1	NM_006225	2
MAGI1	NM_015520	2
NR1I2	NM_033013	2
GATA2	NM_032638	2
ESYT3	NM_031913	2
CLDN11	NM_005602	2
SOX2	NM_003106	2
GP5	NM_004488	2
FAM43A	NM_153690	2
RGS12	NM_198229	2
SOD3	NM_003102	2
NSUN7	NM_024677	2
OCIAD2	NM_152398	2
HOPX	NM_001145459	2
CXCL6	NM_002993	2

BTC	NM_001729	2
POU4F2	NM_004575	2
LRAT	NM_004744	2
ANKRD33B	NM_001164440	2
CCNO	NM_021147	2
TMEM171	NM_001161342	2
OTP	NM_032109	2
PITX1	NM_002653	2
NEUROG1	NM_006161	2
LOC389332	NR_024418	2
EIF4EBP3	NM_003732	2
ANKHD1-EIF4EBP3	NM_020690	2
SLC6A7	NM_014228	2
NKX2-5	NM_001166175	2
HRH2	NM_001131055	2
GRM6	NM_000843	2
SERPINB9	NM_004155	2
HIST1H3C	NM_003531	2
ZNF311	NM_001010877	2
HMGA1	NM_145899	2
MDFI	NM_005586	2
RUNX2	NM_001015051	2
C6orf150	NM_138441	2
AIM1	NM_001624	2
C6orf174	NM_001012279	2
GRM1	NM_001114329	2
LOC441177	NR_027284	2
C6orf176	NR_026861	2
MGC87042	NM_207342	2
IGFBP1	NM_000596	2
PION	NM_017439	2
STEAP4	NM_024636	2
PCOLCE	NM_002593	2
EPO	NM_000799	2
MEST	NM_177524	2
TMEM140	NM_018295	2
PARP12	NM_022750	2
RAB19	NM_001008749	2
PRKAG2	NM_016203	2
DNAJB6	NM_005494	2
NPM2	NM_182795	2
NKX6-3	NM_152568	2
OPRK1	NM_000912	2
RGS20	NM_170587	2
CA3	NM_005181	2
TSPYL5	NM_033512	2

OSR2	NM_053001	2
RGS22	NM_015668	2
C8orf56	NR_027071	2
BAALC	NM_024812	2
OPLAH	NM_017570	2
KIFC2	NM_145754	2
PAX5	NM_016734	2
C9orf122	NR_027294	2
C9orf170	NM_001001709	2
SUSD3	NM_145006	2
BARX1	NM_021570	2
NCRNA00092	NR_024129	2
DAB2IP	NM_032552	2
BARHL1	NM_020064	2
GBGT1	NM_021996	2
ABO	NM_020469	2
DPP7	NM_013379	2
C9orf167	NM_017723	2
AKR1E2	NM_001040177	2
GRID1	NM_017551	2
SLC16A12	NM_213606	2
CNNM1	NM_020348	2
NKX2-3	NM_145285	2
KCNIP2	NM_173195	2
VAX1	NM_199131	2
HMX2	NM_005519	2
EBF3	NM_001005463	2
EFCAB4A	NM_173584	2
KCNQ1DN	NR_024627	2
ST5	NM_213618	2
ALX4	NM_021926	2
SNX32	NM_152760	2
MRGPRF	NM_001098515	2
PDE2A	NM_001143839	2
CCDC67	NM_181645	2
PHLDB1	NM_001144758	2
C1QTNF5	NM_015645	2
MFRP	NM_031433	2
USP2	NM_004205	2
DDX25	NM_013264	2
PUS3	NM_031307	2
TMEM45B	NM_138788	2
ST14	NM_021978	2
WNT5B	NM_030775	2
PDZRN4	NM_001164595	2
PRPH	NM_006262	2

ACVRL1	NM_000020	2
GRASP	NM_181711	2
KRT7	NM_005556	2
DPY19L2	NM_173812	2
C12orf56	NM_001170633	2
KCNC2	NM_139137	2
MAP1LC3B2	NM_001085481	2
NOS1	NM_000620	2
WSB2	NM_018639	2
SRRM4	NM_194286	2
NCOR2	NM_006312	2
GLT1D1	NM_144669	2
FBRSL1	NM_001142641	2
N4BP2L1	NM_001079691	2
KL	NM_004795	2
PCDH17	NM_001040429	2
SLITRK1	NM_052910	2
F7	NM_000131	2
SFTA3	NM_001101341	2
GPR135	NM_022571	2
SLC8A3	NM_182932	2
PAPLN	NM_173462	2
ACOT4	NM_152331	2
BATF	NM_006399	2
GREM1	NM_013372	2
LOC145845	NR_024264	2
DUOXA1	NM_144565	2
IGDCC3	NM_004884	2
ISL2	NM_145805	2
RHCG	NM_016321	2
ANPEP	NM_001150	2
SOCS1	NM_003745	2
SULT1A1	NM_177536	2
C16orf54	NM_175900	2
IRX3	NM_024336	2
HSF4	NM_001040667	2
BHLHA9	NM_001164405	2
SLC13A5	NM_001143838	2
ALOX12B	NM_001139	2
SHISA6	NM_207386	2
CENPV	NM_181716	2
RAB11FIP4	NM_032932	2
SLFN11	NM_001104587	2
PLXDC1	NM_020405	2
PYY	NM_004160	2
NAGS	NM_153006	2

CRHR1	NM_001145148	2
MARCH10	NM_152598	2
GRIN2C	NM_000835	2
FADS6	NM_178128	2
CYGB	NM_134268	2
NPB	NM_148896	2
EMILIN2	NM_032048	2
NOL4	NM_003787	2
ZNF397OS	NM_001166012	2
ONECUT2	NM_004852	2
GALR1	NM_001480	2
SALL3	NM_171999	2
APC2	NM_005883	2
PDE4A	NM_001111308	2
LIPE	NM_005357	2
SYMPK	NM_004819	2
HIF3A	NM_022462	2
MYADM	NM_001020820	2
SLC4A11	NM_032034	2
C20orf103	NM_012261	2
TSPYL3	NR_002781	2
PPP1R16B	NM_015568	2
PTPRT	NM_133170	2
PTGIS	NM_000961	2
NCRNA00176	NR_027687	2
FAM3B	NM_206964	2
MX1	NM_002462	2
GSC2	NM_005315	2
SCARF2	NM_182895	2
NEFH	NM_021076	2
OSM	NM_020530	2
SERHL	NR_027786	2
WNT7B	NM_058238	2
CELSR1	NM_014246	2
ODF3B	NM_001014440	2
TNFRSF25	NM_148965	1
RBP7	NM_052960	1
TNFRSF8	NM_001243	1
MAN1C1	NM_020379	1
C1orf172	NM_152365	1
FGR	NM_005248	1
COL16A1	NM_001856	1
GJB3	NM_001005752	1
GJA4	NM_002060	1
DLGAP3	NM_001080418	1
BEST4	NM_153274	1

FAM159A	NM_001042693	1
TACSTD2	NM_002353	1
FOXD3	NM_012183	1
SGIP1	NM_032291	1
GBP4	NM_052941	1
WNT2B	NM_004185	1
LOC100132111	NR_024237	1
C2CD4D	NM_001136003	1
PCP4L1	NM_001102566	1
FCRLB	NM_001002901	1
LGR6	NM_021636	1
KISS1	NM_002256	1
CR1L	NM_175710	1
GNG4	NM_001098722	1
CGREF1	NM_001166240	1
SPDYA	NM_182756	1
LBH	NM_030915	1
ARHGAP25	NM_001166277	1
VAX2	NM_012476	1
ANKRD53	NM_024933	1
CD8B	NM_172213	1
C2orf55	NM_207362	1
KCNJ3	NM_002239	1
ANKAR	NM_144708	1
TNS1	NM_022648	1
C2orf62	NM_198559	1
FEV	NM_017521	1
MIR375	NR_029867	1
ACCN4	NM_182847	1
B3GNT7	NM_145236	1
C2orf82	NM_206895	1
LOC643387	NR_026923	1
LOC151174	NR_026925	1
MIR149	NR_029702	1
PP14571	NR_024014	1
GPC1	NM_002081	1
KIF1A	NM_004321	1
OXTR	NM_000916	1
CPNE9	NM_153635	1
GHRLOS	NR_024146	1
GHRL	NR_024132	1
RFTN1	NM_015150	1
VILL	NM_015873	1
ZNF662	NM_207404	1
CDCP1	NM_178181	1
USP4	NM_003363	1

C3orf62	NM_198562	1
UBA7	NM_003335	1
SLC38A3	NM_006841	1
CADPS	NM_183394	1
CD200	NM_001004196	1
CCDC37	NM_182628	1
NMNAT3	NM_178177	1
GRK7	NM_139209	1
CHST2	NM_004267	1
SLC7A14	NM_020949	1
ECE2	NM_001037324	1
CPLX1	NM_006651	1
NKX3-2	NM_001189	1
BST1	NM_004334	1
KDR	NM_002253	1
NKX6-1	NM_006168	1
ARHGAP24	NM_001025616	1
SLC6A3	NM_001044	1
IRX4	NM_016358	1
ZBED3	NM_032367	1
LOC645323	NR_015436	1
MEF2C	NM_001131005	1
NR2F1	NM_005654	1
LVRN	NM_173800	1
PRDM6	NM_001136239	1
SHROOM1	NM_133456	1
NME5	NM_003551	1
NRG2	NM_013982	1
PCDHGB5	NM_018925	1
PCDHGC3	NM_002588	1
PCDHGA6	NM_018919	1
PCDHGB4	NM_003736	1
PCDHGA8	NM_032088	1
PCDHGA12	NM_003735	1
PCDHGB3	NM_018924	1
PCDHGA5	NM_018918	1
PCDHGA1	NM_018912	1
PCDHGA11	NM_032092	1
PCDHGA3	NM_018916	1
PCDHGA2	NM_018915	1
PCDHGB6	NM_018926	1
PCDHGC5	NM_018929	1
PCDHGA4	NM_018917	1
PCDHGB2	NM_018923	1
PCDHGA10	NM_018913	1
PCDHGC4	NM_018928	1

PCDHGB7	NM_018927	1
PCDHGA9	NM_018921	1
PCDHGB1	NM_018922	1
PCDHGA7	NM_018920	1
NIPAL4	NM_001099287	1
C5orf52	NM_001145132	1
LCP2	NM_005565	1
FGF18	NM_003862	1
RGS14	NM_006480	1
FOXF2	NM_001452	1
TUBB2B	NM_178012	1
NRN1	NM_016588	1
GCM2	NM_004752	1
GFOD1	NM_018988	1
RBM24	NM_001143942	1
HIST1H4F	NM_003540	1
HIST1H2BH	NM_003524	1
HIST1H3F	NM_021018	1
HIST1H3G	NM_003534	1
HIST1H2BI	NM_003525	1
HIST1H1B	NM_005322	1
HCG9	NR_028032	1
KIAA1949	NM_001134870	1
MICB	NM_005931	1
TULP1	NM_003322	1
TTBK1	NM_032538	1
SPATS1	NM_145026	1
B3GAT2	NM_080742	1
COL12A1	NM_004370	1
GRIK2	NM_001166247	1
VGLL2	NM_182645	1
IL20RA	NM_014432	1
OLIG3	NM_175747	1
SMOC2	NM_022138	1
GRID2IP	NM_001145118	1
TWIST1	NM_000474	1
FERD3L	NM_152898	1
OSBPL3	NM_145321	1
EVX1	NM_001989	1
LOC646999	NR_024390	1
LOC285954	NR_027118	1
AEBP1	NM_001129	1
CCM2	NM_031443	1
CALN1	NM_001017440	1
SRRM3	NM_001110199	1
DPY19L2P4	NR_003551	1

C7orf51	NM_173564	1
LAMB1	NM_002291	1
CLEC2L	NM_001080511	1
EPHA1	NM_005232	1
ATG9B	NM_173681	1
ACCN3	NM_004769	1
SMARCD3	NM_001003802	1
FABP5L3	NR_002935	1
MLL3	NM_170606	1
EN2	NM_001427	1
CNPY1	NM_001103176	1
KBTBD11	NM_014867	1
GATA4	NM_002052	1
LPL	NM_000237	1
SORBS3	NM_005775	1
EGR3	NM_004430	1
PTK2B	NM_004103	1
GPR124	NM_032777	1
STAR	NM_001007243	1
CEBPD	NM_005195	1
NPBWR1	NM_005285	1
CYP7B1	NM_004820	1
FAM135B	NM_015912	1
LYNX1	NM_177457	1
C8orf73	NM_001100878	1
DMRT1	NM_021951	1
MAMDC2	NM_153267	1
GDA	NM_004293	1
GNA14	NM_004297	1
PTGR1	NM_001146109	1
C9orf98	NM_152572	1
ECHDC3	NM_024693	1
RASGEF1A	NM_145313	1
PPYR1	NM_005972	1
PTEN	NM_000314	1
KILLIN	NM_001126049	1
SIGIRR	NM_001135053	1
SCT	NM_021920	1
DRD4	NM_000797	1
EPS8L2	NM_022772	1
TRPC2	NR_002720	1
NRIP3	NM_020645	1
AMPD3	NM_000480	1
DKK3	NM_001018057	1
NAV2	NM_182964	1
LOC100126784	NR_015384	1

AGBL2	NM_024783	1
RTN4RL2	NM_178570	1
BEST1	NM_004183	1
GAL3ST3	NM_033036	1
RIN1	NM_004292	1
TBC1D10C	NM_198517	1
CPT1A	NM_001031847	1
KLHL35	NM_001039548	1
ODZ4	NM_001098816	1
NOX4	NM_001143837	1
C11orf92	NM_207429	1
C11orf93	NM_001136105	1
ESAM	NM_138961	1
ETS1	NM_001143820	1
ACRBP	NM_032489	1
SLC2A14	NM_153449	1
PTHLH	NM_198964	1
DBX2	NM_001004329	1
KCNH3	NM_012284	1
HOXC12	NM_173860	1
NDUFA4L2	NM_020142	1
ARHGAP9	NM_032496	1
MYF6	NM_002469	1
SOCS2	NM_003877	1
MSI1	NM_002442	1
TMEM132D	NM_133448	1
KLHL1	NM_020866	1
ATXN8OS	NR_002717	1
SOX1	NM_005986	1
REC8	NM_001048205	1
SSTR1	NM_001049	1
PTGER2	NM_000956	1
BMP4	NM_130851	1
OTX2OS1	NR_029385	1
LTBP2	NM_000428	1
SLC24A4	NM_153647	1
ATP10A	NM_024490	1
OCA2	NM_000275	1
MEIS2	NM_172316	1
LTK	NM_206961	1
MEGF11	NM_032445	1
PSTPIP1	NM_003978	1
MIR9-3	NR_029692	1
ALDH1A3	NM_000693	1
HBM	NM_001003938	1
RGS11	NM_183337	1

NPW	NM_001099456	1
CCDC64B	NM_001103175	1
ERN2	NM_033266	1
LAT	NM_001014989	1
RRN3P2	NR_003369	1
FAM57B	NM_031478	1
IRX5	NM_005853	1
MT1H	NM_005951	1
FBXL8	NM_018378	1
FAM65A	NM_024519	1
CLEC18A	NM_182619	1
CLEC18C	NM_173619	1
ATP2C2	NM_014861	1
CBFA2T3	NM_175931	1
DOC2B	NM_003585	1
P2RX5	NM_175080	1
ALOX15	NM_001140	1
VMO1	NM_001144940	1
ZFP3	NM_153018	1
BCL6B	NM_181844	1
KCNAB3	NM_004732	1
GUCY2D	NM_000180	1
ABHD15	NM_198147	1
CORO6	NM_032854	1
LHX1	NM_005568	1
ARL5C	NM_001143968	1
RAPGEFL1	NM_016339	1
IGFBP4	NM_001552	1
C1QL1	NM_006688	1
LOC100128977	NR_024559	1
C17orf57	NM_152347	1
SKAP1	NM_001075099	1
PRAC	NM_032391	1
C17orf93	NR_024103	1
HOXB13	NM_006361	1
SAMD14	NM_174920	1
MIR142	NR_029683	1
C17orf64	NM_181707	1
LIMD2	NM_030576	1
ICAM2	NM_001099786	1
ST6GALNAC2	NM_006456	1
MGAT5B	NM_144677	1
LOC283999	NM_001145529	1
SLC16A3	NM_001042422	1
L3MBTL4	NM_173464	1
SLMO1	NM_006553	1

KCTD1	NM_001136205	1
RAX	NM_013435	1
AMH	NM_000479	1
JSRP1	NM_144616	1
LINGO3	NM_001101391	1
NFIC	NM_205843	1
TICAM1	NM_182919	1
VAV1	NM_005428	1
CLEC4GP1	NR_002931	1
ICAM1	NM_000201	1
S1PR5	NM_001166215	1
ZNF763	NM_001012753	1
HPN	NM_182983	1
NFKBID	NM_139239	1
GMFG	NM_004877	1
DLL3	NM_203486	1
CNTD2	NM_024877	1
POU2F2	NM_002698	1
ZNF575	NM_174945	1
PNMAL2	NM_020709	1
PRKD2	NM_001079882	1
SLC8A2	NM_015063	1
FUT1	NM_000148	1
SLC17A7	NM_020309	1
MYH14	NM_001145809	1
ZNF578	NM_001099694	1
ZNF677	NM_182609	1
CACNG8	NM_031895	1
SCRT2	NM_033129	1
EBF4	NM_001110514	1
OXT	NM_000915	1
SPEF1	NM_015417	1
LRRN4	NM_152611	1
CD93	NM_012072	1
KCNK15	NM_022358	1
STX16	NM_001134773	1
CTSZ	NM_001336	1
RTEL1	NM_016434	1
IFNGR2	NM_005534	1
DSCAM	NM_001389	1
TMPRSS2	NM_001135099	1
C21orf57	NM_058181	1
TUBA8	NM_018943	1
sept-05	NM_002688	1
ELFN2	NM_052906	1
TNFRSF13C	NM_052945	1

PKDREJ	NM_006071	1
ADM2	NM_024866	1
